# How does owning commercial housing affect the subjective well-being of rural-urban migrants?——The mediating effect of housing assets and the moderating effect of debt

**DOI:** 10.1371/journal.pone.0287258

**Published:** 2023-06-28

**Authors:** Wenlong Lou, Cuicui Du, Yuhua Qiao

**Affiliations:** 1 School of Public Management, Yanshan University, Qinhuangdao, Hebei, China; 2 Key Research Bases of Humanities and Social Sciences for Universities in Hebei Province—Yanshan University County Area Revitalization Development Policy Research Centre, Qinhuangdao, Hebei, China; 3 Political Science Department, Missouri State University, Springfield, Missouri, United States of America; Zhejiang University of Finance & Economics, CHINA

## Abstract

Houses mean a lot to Chinese people, and in the context of the urban-rural dualist system, town housing has a special significance for rural-urban migrants. Based on the 2017 China Household Finance Survey(CHFS) data, this study uses the Ordered Logit (OLogit) model to empirically test the effect of owning commercial housing on the subjective well-being(SWB) of rural-urban migrants, and through the mediating effect and moderating effect to conduct an in-depth investigation into the intrinsic effect mechanism and further explains the relationship between the two and the current residential location of their family. The results of the study show that: (1) Owning commercial housing can significantly enhances the subjective well-being(SWB) of rural-urban migrants, and the findings remain robust after using alternative model, adjusting the sample size, correcting for sample selectivity bias using propensity score matching(PSM), and controlling for potential endogeneity bias combining instrumental variables and conditional mixed process(CMP); (2) The effect of owning commercial housing on the subjective well-being(SWB) of the first generation rural-urban migrants, rural-urban migrants in the eastern and central regions, and those who obtained housing before the rapid rise in house prices is more pronounced; (3) Commercial housing acts on the subjective well-being(SWB) of rural-urban migrants through the mediating effect of housing assets, and there is some regional variation in the mediating effect of housing assets. At the same time, the household debt acts as a positive moderator between commercial housing and the subjective well-being(SWB) of rural-urban migrants; (4) Even with commercial housing, rural-urban migrants whose families are currently living in rural areas still have a stronger sense of subjective well-being (SWB).

## 1 Introduction

Well-being is everyone’s aspiration for a better life. It has become a major indicator of quality of people’s livelihoods [1], gradually replacing the traditional GDP, and an important goal for our government to focus on. At the 19th National Congress of the Communist Party of China, it was stated that the original intention and mission of the Chinese Communists is to work for the happiness of the Chinese people and the rejuvenation of the Chinese nation. After the reform and opening up, a large number of rural-urban migrants entered cities to engage in non-agricultural work in order to seek higher income, which also created a trend of massive rural exodus. The number of rural-urban migrants in China has reached a peak of 296 million in 2022 [2]. While hundreds of millions of rural-urban migrants have made enormous contributions to China’s economic development, how to enhance the SWB of this huge group is not only a solemn commitment of the Party and the government, but also the future direction of our social development. Therefore, the SWB of rural-urban migrants is an important research topic.

With the introduction of the "Easterlin paradox", more scholars began to explore happiness, thus creating a new field of economics: the economics of happiness. At present, domestic and foreign scholars’ research on happiness mainly focuses on four aspects: economic factors, political factors, socio-demographic factors and environmental factors. In recent years, global housing prices have been rising, and coupled with the fact that housing issues affect the national economy and people’s livelihood, more and more studies have begun to focus on the impact of housing on people’s SWB, mainly in terms of housing ownership [3–8], housing quantity and area [8, 9], housing prices [8–11] (or housing pressure [12, 13]), housing security [14], and residential community [15]. In China, there is almost no housing market in rural areas, so the research on the relationship between housing and SWB mainly focuses on all residents or urban residents. Even if the floating population is involved [11, 15, 16], few studies focus on rural-urban migrants to explore the impact of town commercial housing on their SWB, which results in the incompleteness of the research subjects. Although the core view of a large number of domestic and foreign studies is that housing ownership has a significant positive impact on SWB, this view has not been rigorously confirmed for low-income people [17]. Rural-urban migrants are a representative low-income group in China, and their numbers are increasing year by year. They have been moving from rural areas to cities and towns and are also eager to settle down here. In fact, there are some rural-urban migrants who already own commercial housing in towns or cities, but in general, due to their low level of education and limited income, the difficulty and significance of owning commercial housing in towns or cities are different from those of local residents or urban-urban migrants. Then, can owning commercial housing improve the SWB of these groups? It is worth noting that there is the large house price gap among the east, the central, and the west, the consumption philosophies differ between the first and second generation of rural-urban migrants, and rapid changes in China’s housing market. we also need to analyze the heterogeneity of the impact of owning commercial housing on the SWB of rural-urban migrants from the perspective of intergenerational, regional and housing acquisition time.

In addition, academics have summarized several possible channels through which housing affects the SWB of residents, including household asset portfolio and level of wealth accumulation [18], community activities [19], child development and quality of education [20], physical health [21], psychological feelings [22], quality of life [23], perceived living standards [24] and flexibility (or mobility) constraints and precautionary savings [25]. But the analysis of the mechanisms underlying the influence of housing assets and household debt is not sufficiently in-depth. For rural-urban migrants with lower income levels and a desire to own a house in the town, the difference in the commercial housing owned may also have a differential impact on the SWB of rural-urban migrants. On the one hand, the higher the price, the higher asset value which may lead to higher SWB for rural-urban migrants. On the other hand, the higher price may also mean higher debt burden, which may reduce their SWB. So, do they get a greater wealth effect or a greater debt burden from owning commercial housing? And how does this indirectly affect the SWB of rural-urban migrants? Is there a regional difference in this underlying mechanism?

More importantly, the location of urban residents’ commercial housing is mostly the current residence of their families, and their main economic and social activities are also in the urban. However, for rural-urban migrants, they are still in the state of "peri-urbanization". Even if they have commercial housing, the main members of their families may still live in rural areas, which is obviously different from urban residents. So what is the relationship between commercial housing, the current residential location of the family and the SWB of rural-urban migrants? This question has not been answered in detail.

To answer these research questions, this paper uses data from the 2017 China Household Finance Survey (CHFS) to empirically test the effect of owning town commercial housing on the SWB of rural-urban migrants. This research makes four possible contributions to the literature. First, the research perspective is novel. This study focuses on the impact of owning commercial housing on the SWB of rural-urban migrants, enriching the existing literature on housing and the SWB of residents, complementing the research sample of the group of rural-urban migrants, verifying whether owning commercial housing by low-income groups such as migrant workers can still improve their SWB, and making a complete chain of evidence available for empirical research on the topic of housing and SWB; Second, the empirical strategy is rigorous. This study uses propensity score matching (PSM) to correct for selectivity bias and combines instrumental variables and conditional mixed process (CMP) to better overcome potential endogeneity bias, ultimately yielding robust analytical results; Third, the underlying impact mechanism is explored. From the perspective of housing assets and household debt, this study also focuses on analyzing the underlying mechanism of the effect of commercial housing on the SWB of rural-urban migrants and the regional differences in such mechanism; Fourth, this study also further discusses the relationship between commercial housing, current residential location of their family and the SWB of rural-urban migrants in China’s “semi-urbanization” state, and finds that they have not completed the family-oriented migration.

## 2 Theoretical analysis and research hypotheses

Research on the relationship between homeownership and the SWB has been receiving extensive attention from scholars across countries. A large body of research literature has concluded that homeownership can enhance the SWB of residents [3–7]. Fong et al. proposed that the impact of homeownership on happiness should fully consider the socio-demographic background, and an important socio-demographic factor is migration status [26]. Under the dual system of urban and rural areas in China, housing not only serves as a place to live, but also determines the objects of social interaction, leading to social stratification. Zhang and Yang found that, in addition to the conventional criteria of class stratification such as income, education, and occupation, homeownership has become a major determinant of subjective class identification in China [27]. Homeowner status is an indicator of one’s financial resources that reflect one’s social status. A high socioeconomic status or a much peer-admired sociometric status may make one happy [28, 29].

Rural-urban migrants are a unique type of floating population in China. Rural-urban migrants who own a home in an urban area are significantly happier than those who do not. Those who own a home in a rural area are not significantly happier than those that do not [26]. Given the income they make, with house prices continuing to rise it is more difficult for rural urban migrants to buy a home in the big cities where they have moved to. As a result, returning home to buy a house, that is, buying commercial housing in towns, has become another option for many rural-urban migrants in recent years. First of all, rural-urban migrants owning a commercial home in the town means a transition from being a renter to a homeowner, which leads to a significant improvement in their psychological wellbeing [30], a sense of accomplishment in achieving important life goals, and thus a greater sense of the SWB; Second, compared to rural areas, commercial housing in towns represents better social welfare and a higher level of public services, including medical resources, educational resources for children, etc., thus improving the overall quality of life of rural-urban migrants and enhancing their sense of the SWB; Once more, compared with renting a house, owning commercial housing is more helpful for rural-urban migrants to participate in community affairs, get in touch with local residents, and accumulate social capital [30], so that rural-urban migrants can integrate into the city more quickly and improve their sense of happiness. Last but not least, in an increasingly competitive marriage market due to a rising sex ratio imbalance, commercial housing has even become a prerequisite for marriage in many regions of China [31, 32]. And these phenomena are more obvious in rural areas, most women are reluctant to marry back to the countryside after moving to the city, and the marriage requirements of women who remain in the village are getting higher and higher. Therefore, owning commercial housing can improve the chances of male rural-urban migrants or their sons winning the competitive marriage market, and owning a house can reduce the divorce rate in the family, both of which greatly improve their SWB [30]. To sum up, the first hypothesis is proposed as follows:

**Hypothesis 1.** *Rural-urban migrants who own commercial housing have stronger SWB than those who do not*.

Housing is characterised by multiple attributes, both residential (use value) and investment (exchange value). In terms of asset attributes, housing as a fixed asset [33], housing equity tends to be the largest component of a household’s wealth portfolio [34], accounting for about 60% to 80% of the total assets [35]. Both the ownership of a home, which increases the amount of asset, and the increase in the price of a home, which appreciates the value of asset [36], can significantly enhance one’s SWB. Homeownership is not only an indicator of wealth, but also an effective means of accumulating wealth [26], and more housing wealth increases the SWB [37, 38]. To sum up, the second hypothesis is proposed as follows:

**Hypothesis 2.** *Commercial housing affects the SWB of rural-urban migrants through housing assets*. *That is*, *housing assets play a partial mediating effect*.

Debt has both positive and negative effects on the SWB. On the one hand, according to the liquidity constraint theory, when households face liquidity constraints (such as a large housing expense), borrowing can smooth out current consumption, thereby improving the satisfaction of current economic life and thus increasing one’s sense of the SWB [39]. On the other hand, a report in 2018 [40] suggested that China’s households have a high level of debt. Between January and October 2017, the Chinese household leverage rose from 44.8 to 53.2%. Xiao et al. finds that any type of debt holding is negatively associated with happiness [41]. For rural-urban migrants, the high cost of housing in China often means that owning a commercial home comes with a corresponding financial burden [42], such as housing loans [43], which may lead to some households having to cut other expenses to pay the mortgage. Therefore, in theory, debt is highly likely to reduce people’s SWB by restraining consumption [38]. Balmer et al. concludes that heavy repayment pressure can squeeze one out of spending on "health", such as those who owe money choose cheaper medicines or eat junk food [44]. Additionally, the stress of repaying debt may also be detrimental to the psychological wellbeing of those who owe a large amount of money and thus affect their SWB [45]. To sum up, the third hypothesis is proposed as follows:

**Hypothesis 3.** *The SWB of rural-urban migrants who own commercial housing is affected by the moderating effect of household debt*.

People in the Western world generally report lower levels of SWB in urban areas than in rural areas [46]. Further, from the perspective of rural-urban migration in developing countries, Knight and Gunatilaka finds that rural-urban migrant households which have settled in urban China report lower happiness than rural households, despite their higher mean income [47]. China’s urbanization process is still in a state of "peri-urbanization", and the rate of citizenship of rural migrants in China is much slower than the rate of migration to the towns, and the agricultural migrants have not really integrated into the cities either socially or psychologically after entering the cities [48]. This leads to a situation where even if the rural-urban migrants own commercial housing in the town, it is easy to form a phenomenon of separation of housing and residence, or the rural-urban migrants have not completed family migration, in short, they would feel happier if their family lived in the rural areas. To sum up, this paper continues to extend the analysis of the impact of the current residential location of households of rural-urban migrants who have owned commercial housing on their SWB, and the fourth hypothesis is proposed as follows:

**Hypothesis 4.** *After owning commercial housing*, *compared with towns*, *rural-urban migrants whose family’s current residential location is in rural areas still have a greater sense of SWB*.

**Fig 1:**
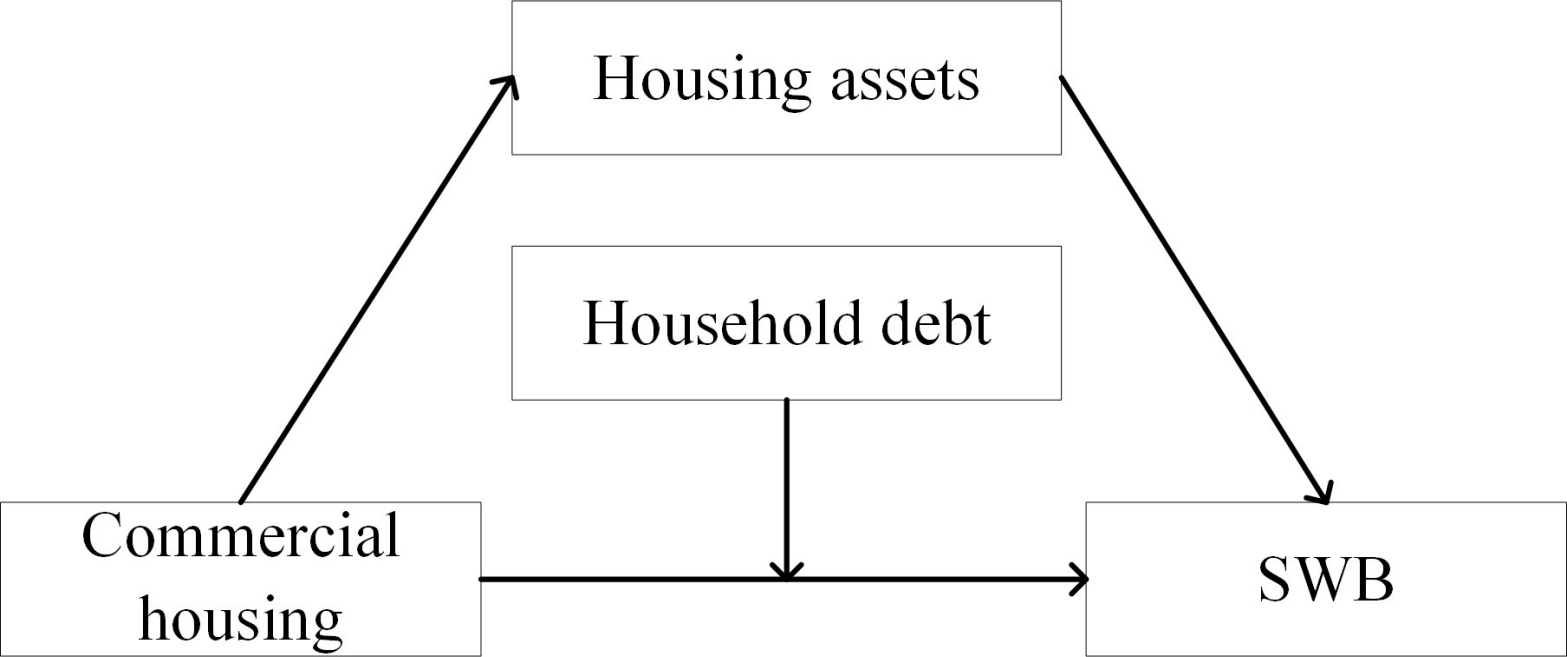
Path diagram of the impact of commercial housing on the SWB of rural-urban migrants.

Based on the above assumptions, a path analysis diagram of the impact of commercial housing on the SWB of rural-urban migrants is drawn, as shown in Fig 1.

## 3 Data, variables and methods

### 3.1 Data

The China Household Finance Survey (CHFS) is a nationwide sample survey project conducted by the China Household Finance Survey and Research Centre of Southwestern University of Finance and Economics. The baseline survey was launched in 2011 and four follow-up surveys have been successfully implemented in 2013, 2015, 2017 and 2019 respectively. Using a stratified three-stage probability proportion to size (PPS) random sampling method, the CHFS data includes information related to housing assets and financial wealth, debt and credit constraints, income and consumption, social security and insurance, demographic characteristics and employment, and subjective attitudes, filling a gap in academic research and the current state of household finance in China, with a high degree of data representativeness. This study uses 2017 CHFS data (The latest round of CHFS data is conducted in 2019, but based on the purpose of the study and variable screening, the variable setting in 2019 is not as comprehensive as that in 2017, and the final sample size obtained is relatively small, both of which may lead to bias in the estimation results; therefore, we use 2017 data for the study.), and the survey sample covers 29 provinces (autonomous regions and municipalities directly under the central government), 355 counties (districts and county-level municipalities), and 1428 village (neighborhood) committees across China, with a sample size of 40011 households and 127012 individuals. According to the research purpose, this paper merged family data and personal data. We have particular interest in the relationship between commercial housing and the SWB of rural-urban migrants. Using counties (districts and county-level municipalities) as a unit, individuals without local *hukou* are considered to be migrants, and we define migrants with rural *hukou* elsewhere as rural-urban migrants. On this basis, the rural-urban migrants who owned their houses were retained, and samples with missing and invalid values in the variables were eliminated. The remaining sample size becomes 5477. In addition, provincial (municipality) data from the statistical database of China Economic Network.

Researchers have carried out a lot of research on the relationship between housing and SWB. And there are many statistical databases that can measure the variable of housing and SWB, including Chinese Social Survey (CSS), Chinese General Social Survey (CGSS), China Migrants Dynamic Survey (CMDS), China Household Finance Survey (CHFS), etc. However, in addition to caring about how commercial housing affects the SWB of rural-urban migrants, we also focus on exploring the underlying mechanism from the perspective of housing assets and household debt and expanding the research on current family residential location and SWB of rural-urban migrants with commercial housing in this study. The advantage of the CHFS database is that it not only includes the dependent variable of SWB, the independent variable of commercial housing, but also has many other related required variables, such as the demographic characteristics, household characteristics, socio-economic characteristics, social security and subjective attitude. And more importantly, it has the rich information in terms of assets and debts, which provides important data support for our research purposes. Therefore, we choose CHFS data for research.

### 3.2 Variables

#### 3.2.1 Dependent variables

Subjective well‑being (SWB). The CHFS questionnaire asks respondents "In general, do you feel happy now?" Respondents were given a five-point Likert scale of integers from 1 to 5, representing "very happy", "happy", "neutral", "unhappy" and "very unhappy". Based on the needs of the study, they were reassigned to a scale from 1 to 5 to represent "very unhappy" to "very happy" respectively. The survey results show that the average SWB of rural-urban migrants is 3.84, which is between neutral and happy, 49.06% of the samples answered happy, and 20.18% answered very happy. Overall, 69.24% of rural-urban migrants considers themselves happy (as shown in Table 1). This SWB measurement method can truly reflect the SWB of rural-urban migrants, thereby making the SWB of different individuals comparable [49].

**Table 1:** Statistics on SWB of rural-urban migrants.

SWB score	1	2	3	4	5	Total	Mean
Sample size	44	210	1431	2687	1105	5477	3.84
Proportion (%)	0.80	3.83	26.13	49.06	20.18	100	——

#### 3.2.2 Independent variables

Whether owning commercial housing (Commercial housing in this study refers to the ownership of commercial housing by rural-urban migrants. In China, commercial housing refers to houses developed by real estate development and operation companies, approved by the relevant government departments, and built for sale and rental in the market, and the commercial housing has the property rights certificate and the land certificate. In contrast, self-occupied residential houses built by oneself, participation in or commissioned construction on state-owned land and collective land do not fall within the scope of commercial housing. In the context of China’s urban-rural dualist system, commercial housing exists only in urban or town areas and needs to be acquired by purchase, while there is almost no housing market in rural areas, and the houses they live in are collectively allocated homestead and are not available for sale. Importantly, owning commercial housing means that rural-urban migrants can improve their living standards, enjoy better public service resources such as healthcare and education in towns and cities, and can improve their competitiveness in the fierce marriage market.). The CHFS questionnaire asks the respondents about their housing situation, the value for this variable is based on the questions "How was the house acquired?" and "What is the form of ownership of this house?" to determine whether a rural-urban migrant owns commercial housing. Rural-urban migrants who answered "buying new commercial housing" or "buying second-hand commercial housing" and those who answered "property form is commodity housing" will be assigned a value of 1, otherwise they will be assigned a value of 0 (For the first question, there are eight other options: purchasing policy housing (affordable housing, capped-price housing, etc.), inheritance or donation, purchasing from unit below market price (housing-reform house, welfare housing, etc), fund-rasing building, self-establish/expansion, settlement housing (compensation for collection of land), purchasing houses with limited property rights and Others. And the second question has seven other options: policy housing (affordable housing, capped-price housing, self-using commercial housing), purchased public housing (public housing whose property belongs to military, units in Beijing or the local), housing for using only (public housing controlled by government or units), house-site housing on collective-owned land, houses with limited property rights (on collective-owned land), commercial & residential house and Others. These answers are all considered as not owning commercial housing.). The survey results show that the proportion of rural-urban migrants owning commercial housing is 35.5%.

#### 3.2.3 Mediator variables

Housing assets. Mediating effect refers to the effect of the independent variable (X) on the dependent variable (Y) through the mediator variable (M) [50]. This paper employs the total housing assets of rural-urban migrants’ households as the mediator variable to explain the impact mechanism of whether rural-urban migrants own commercial housing on their SWB. The questionnaire asks respondents "How much is this house worth at the moment?" and this question is employed to totalize each housing asset owned by a rural-urban migrant household to obtain a total housing asset value for each household, which is incorporated into the model in logarithmic form.

#### 3.2.4 Moderator variables

Household debt. Moderating effect means that the effect of the independent variable (X) on the dependent variable (Y) is moderated by the moderator variable (M) [51]. This paper employs whether rural-urban migrants’ families have debt as the moderator variable to investigate whether the impact of rural-urban migrants’ ownership of commercial housing on SWB is moderated by household debt. A value of 1 is assigned to those who have household debt, otherwise a value of 0 is assigned.

#### 3.2.5 Control variables

To ensure the scientific rigour of the study, on the basis of the existing research literature [47, 52–55], the demographic characteristics, household characteristics, socio-economic characteristics, social security and subjective attitude of the respondents were selected for inclusion in the model as control variables affecting the SWB of rural-urban migrants. Demographic characteristics include gender, age, education and subjective health status; Household characteristics include head of household, marital status and current residential location of the family. Marital status is divided into "married" and "single". The former includes "married", "cohabiting" and "remarried" and is assigned a value of 1, and the latter includes "unmarried", "separated", "divorced" and "widowed" and is assigned a value of 0 [38]. Moreover, the questionnaire counts the location where the respondent households currently live. The options include "urban area of a large city", "suburban area of a large city", "large town", "small town", "township" and "rural area", with the first four defined as "town" and assigned a value of 1, and the last two defined as "rural area" and assigned a value of 0; Socio-economic characteristics include job type, annual income per capita and annual daily expenditure per capita; Social security refers to the possession of health insurance for rural-urban migrants; Social attitude refers to the extent to which respondents trust people they do not know, with "fairly trusting" and "very trusting" defined as trusting and assigned a value of 1, and "very distrustful", "less trusting" and "neutral" defined as distrustful and assigned a value of 0. Moreover, perGDP and population scale were added as control variables for provincial (municipality) characteristics. In addition, given the heterogeneity of the impact of commercial housing on rural-urban migrants in different regions, dummy variable is employed to control the regional effect, which is divided into eastern, central and western regions. The detailed meaning and descriptive statistics of each variable are shown in Table 2.

**Table 2:** Descriptive statistical analysis of variables.

Variable	Variable name	Definition	Mean	SD
Dependent variable	SWB	1 = very unhappy; 2 = unhappy; 3 = neutral; 4 = happy; 5 = very happy	3.840	0.814
Independent Variable	Commercial housing	Whether to own commercial housing: 1 = yes; 0 = no	0.355	0.479
Mediator Variable	Housing assets	Logarithm of total household housing assets	9.937	5.150
Moderator variable	Household debt	Whether there is household debt: 1 = yes; 0 = no	0.481	0.500
Control Variables	Gender	1 = male; 0 = female	0.600	0.490
	Generation	1 = rural-urban migrants born in 1980 and later (the second generation); 0 = rural-urban migrants born before 1980 (the first generation)	0.570	0.495
	Education	1 = no education; 2 = primary school; 3 = junior high school; 4 = senior high school or technical school; 5 = college; 6 = undergraduate (Bachelor degree); 7 = postgraduate (Master degree); 8 = postgraduate (Doctor degree)	3.331	1.213
	Subjective health status	1 = very poor; 2 = poor; 3 = neutral; 4 = good; 5 = very good	3.817	0.900
	Head	1 = yes; 0 = no	0.307	0.461
	Marital status	1 = married; 0 = single	0.751	0.432
	Residential location	1 = town; 0 = rural area	0.525	0.499
	Job type	1 = governmental department and organization/public institution or state-owned enterprise (SOE)/state holding enterprise; 0 = otherwise	0.093	0.291
	Per-income	Logarithm of annual household income per capita (Yuan)	9.511	1.709
	Per-expenditure	Logarithm of annual per capita household expenditure on water, electricity, fuel, property management, heating, etc. (Yuan)	6.298	1.363
	Social security	Whether there is social medical insurance: 1 = yes; 0 = no	0.894	0.308
	Social attitude	1 = trustful; 0 = distrustful	0.056	0.229
	PerGDP	Logarithm of per capita GDP of each province (municipality) in 2017 (Yuan)	10.955	0.340
	Population scale	Logarithm of the permanent population of each province (municipality) in 2017 (Ten thousand people)	8.534	0.663

### 3.3 Methods

#### 3.3.1 Baseline model——Ordered logit model

The SWB of rural-urban migrants is a typical discrete ordinal variable. This study therefore employs the ordered logit model to empirically analyse the effects of commercial housing on SWB of rural-urban migrants. The Ologit model setting in this study is shown in Formula (1):

$$SWBB_{i}=\alpha +\beta Commercial\;Hou\sin g_{i}+\gamma Z_{i}+\delta Area_{i}+\varepsilon_{i}$$ (1)

Where *SWB*_*i*_ denotes the SWB of the ith rural-urban migrant; *Commercial Hou*sin*g*_*i*_ denotes whether the rural-urban migrant owns commercial housing; *Z*_*i*_ indicates control variables; *Area*_*i*_ denotes regional dummy variable; *α*、*β*、*γ* and *δ* denote theparameters to be estimated and *ε*_*i*_ denotes stochastic disturbance team.

#### 3.3.2 Correcting selection bias——Propensity Score Matching (PSM)

There is a consideration that whether a rural-urban migrant owns commercial housing may not satisfy random sampling, but a self-selection process determined by individual characteristics. That is, systematic differences in initial conditions (rural-urban migrants’ own endowments, such as age, health, marital status, etc.) other than commercial "housing" If that is the case, direct regression may result in selectivity bias due to non-random sampling. For this reason, this study uses propensity score matching (PSM) to construct a counterfactual framework to correct for this.

The Propensity Score, first proposed by Rosenbaum and Rubin [56], is the conditional probability of assignment to a particular treatment given a vector of observed covariates. The propensity score allows the effect of covariates to be excluded, giving a net effect between independent variable and dependent variable. We set the binary dummy variable *D*_*i*_ = {0, 1} to indicate whether the rural-urban migrant owns commercial housing. In our research, *D*_*i*_ = 1 thus denotes that the rural-urban migrant own commercial housing, and *D*_*i*_ =0 denotes that he do not. And there may be two states for the SWB (*y*_*i*_) of rural-urban migrants, *y*_1*i*_ indicates the SWB of rural-urban migrants who own commercial housing, while *y*_0*i*_ indicates the SWB of rural-urban migrants who do not own commercial housing.

The principle of the propensity score matching (PSM) method is to use the logit model to calculate the propensity score p, also known as the probability p, of rural-urban migrants owning commercial housing based on observable covariates. The treatment and control groups are then matched according to p-score by different matching methods (k-nearest neighbor matching, radius matching, kernel matching, local linear regression matching), thus eliminating sample selectivity bias and acting as a random experiment. Finally, the difference between the SWB of rural-urban migrants who own commercial housing (i.e. *E*(*y*_1*i*_|*D*_*i*_ = 1)) and the SWB of rural-urban migrants who do not (i.e. *E*(*y*_0*i*_|*D*_*i*_ = 1)) is calculated on the basis of satisfying the balance test and overlap assumption, that is, the value of ATT (Average Treatment Effect on the Treated). As shown in Eq (2).


$$ATT_{PSM}=E(y_{1i}-y_{0i}|D_{i}=1)=E(y_{1i}|D_{i}=1)-E(y_{0i}|D_{i}=1)$$ (2)

In fact, *E*(*y*_0*i*_|*D*_*i*_ = 1) cannot be observed, and the advantage of the PSM method is to find an effective control group *E*(*y*_0*i*_|*D*_*i*_ = 0) to replace *E*(*y*_0*i*_|*D*_*i*_ = 1) for rural-urban migrants who actually own commercial housing, so as to achieve "counterfactual" estimation.

#### 3.3.3 Instrumental variables method

The propensity score matching (PSM) method can correct the selection bias caused by observable variables, but this study may also have endogeneity problems caused by omitted variables and reverse causality. Firstly, factors that are difficult to measure, such as the personal experience or psychological feelings of rural-urban migrants, may also affect their SWB, leading to the problem of missing variables; Secondly, the SWB of rural-urban migrants may affect their probability of owning commercial housing, that is to say, rural-urban migrants who are happier are more likely to live in towns and thus settle in towns, and are more likely to own commercial housing. Conversely, rural-urban migrants with a low sense of SWB may choose to return to rural areas and are less likely to own commercial housing. This creates a reverse causality problem.

Potential endogeneity issues will lead to inconsistent estimated coefficients, which are commonly addressed by the instrumental variables method. Most current studies use higher area-level housing characteristics variables as instrumental variables for individual housing variables. In line with the relevant literature [57, 58] and characteristics of the data, this paper selects the variable of the commercial housing ownership rate of prefecture-level cities in the sixth national census data as an instrumental variable (IV1). At the same time, considering that the research object of this paper is the rural-urban migrant group, the variable of the commercial housing ownership rate of the rural population in each region is further selected as the instrumental variable (IV2) (The reasons and advantages of using this data as an instrumental variable lie in two aspects: Firstly, the county-by-county data of the sixth census involves housing data of various county-level cities across the country, and the regional housing ownership rate data calculated according to the census should be the current relatively comprehensive and authoritative survey results; Secondly, the influence of macro variables on individual behavioral decision-making tends to be lagged, and since the "the sixth census data" has a lag of several years from the survey year of the sample data in this paper, which avoids the possible mutual influence between the independent variables and the instrumental variables, and exogenous conditions are easier to satisfy.). On the one hand, the regional commercial housing ownership rate will affect the probability of households owning commercial housing, that is, the instrumental variables and endogenous variable meet the correlation conditions. On the other hand, the regional commercial housing ownership rate does not directly affect the individual SWB of rural-urban migrants, which indicates that the instrumental variables (IV1 and IV2) satisfy the exogenous condition.

In this paper, since the dependent variable of SWB is ordinal, and the endogenous variable of commercial housing is binary, neither two stage least square (2SLS) nor binary selection model (IV-Probit) is applicable. In order to estimate the instrumental variable model more effectively, we employ the conditional mixed process (CMP) to test for endogeneity. Referring to Roodman [59], the CMP method has a strong coverage of the variable categories of the dependent variables and endogenous variables, and thus different models can be selected for estimation. The CMP method is also divided into two stages. In our research, the first stage employs the oprobit model (ordered probit model) to estimate the effect of owning commercial housing on SWB. In the second stage, commercial housing is used as the dependent variable, and the instrumental variable as the independent variable, and the probit model is used to estimate.

#### 3.3.4 Analysis of impact mechanisms

*3*.*3*.*4*.*1 Mediating effect model*. The traditional procedure for testing mediating effects is the stepwise regression method proposed by Baron and Kenny [60] (BK test method for short), which has since been further developed by other researchers with the Sobel test [61] and the Bootstrap test [62]. However, these methods are mainly applicable to mediating effect models of continuous variables. In this paper the dependent variable of SWB (Y) is ordinal and the mediator variable (M) of housing assets is continuous. That is, the regression coefficient of M on X (a measure of the continuous variable) is not on the same scale as both the regression coefficient of Y on M (a logit measure) and the regression coefficient of Y on X (a logit measure), so the method of testing the mediating effect of the continuous variable cannot simply be used. Therefore, We employ the method proposed by Karlson, Holm and Breen [63] (KHB method for short), which can decompose the effects of discrete variables and continuous variables, so as to test the mediating effect of the cross-model. The stepwise method and Bootstrap test were also used to test for robustness (treating the dependent variable of SWB as a continuous variable). The set of equations for the mediating effect is shown in Eqs (1) and (3)–(4).


$$Hou\sin g\;assets_{i}=\alpha +\beta Commercial\;Houseing_{i}+\gamma Z_{i}+\delta Area_{i}+\varepsilon_{i}$$ (3)

$$SWB_{i}=\alpha +\beta Commercial\;Hou\sin g_{i}+\varphi Hou\sin g\;assets_{i}+\gamma Z_{i}+\delta Area_{i}+\varepsilon_{i}$$ (4)

Where *Hou*sin*g assets*_*i*_ denotes household housing assets as the mediator variable; *Commercial Hou*sin*g*_*i*_ denotes independent variable; *α*、*β*、*γ*、*φ* and *δ* denote the parameters to be estimated, and the rest is consistent with the baseline model.

*3*.*3*.*4*.*2 Moderating effect model*. To test whether there is a moderating effect of household debt on the impact of commercial housing on the SWB of rural-urban migrants, a regression model is further constructed as shown in Eq (5).


$$SWB_{i}=\alpha +\beta Commercial\;Hou\sin g_{i}+\eta Debt_{i}+\theta Commercial\;Hou\sin g\times Debt_{i}+\gamma Z_{i}+\delta Area_{i}+\varepsilon_{i}$$ (5)

Where *Debt*_*i*_ denotes household debt as the moderator variable; *Commercial Hou*sin*g*_*i*_×*Debt*_*i*_denotes the interaction term; *α*、*β*、*η*、*θ*、*γ* and *δ* denote the parameters to be estimated, and the rest is consistent with the baseline model. We focus on the interaction term coefficient (*θ*), if *θ* = 0 means that the moderating effect is not present. If debt can strengthen the effect of commercial housing on the SWB of rural-urban migrants, then *θ*>0, otherwise, *θ*<0.

## 4 Empirical findings

This paper uses the software Stata14 for empirical analysis, the goal is to explore whether commercial housing ownership can improve the SWB of rural-urban migrants. First, with commercial housing as the independent variable, the Ologit model is used to estimate the impact of commercial housing on SWB; Then, the robustness of the estimated results is tested by replacing the model (replacing the Ologit model with the Oprobit model and OLS model) and adjusting the sample size. Moreover, considering that rural-urban migrants owning commercial housing may be the result of "self-selection", the PSM method is used to correct the selection bias for this reason, and the CMP method is used to test other endogeneity issues; Next, this study further analyses the heterogeneity of the impact of commercial housing ownership on the SWB of rural-urban migrants from two perspectives: intergenerational and regional differences; After this, in order to examine the mechanism of the effect of owningcommercial housing on the SWB of rural-urban migrants, a model of the mediating effect of housing assets and a model of the moderating effect of household debt are constructed to verify this. Lastly, we continues to extend the analysis of the impact of the current residential location of households of rural-urban migrants who have owned commercial housing on their SWB.

### 4.1 Baseline regression results

Table 3 shows the estimation results of models 1–3 for the ordered logit regression. Where model 1 only controls for independent variable, model 2 adds control variables and model 3 further incorporates regional dummy variable into the model. Overall, the Pseudo R^2^ value gradually increase from model 1 to model 3, and the χ^2^ value all pass the significance test at the 1% statistical level, and there is no significant change in the direction of influence and significance level of the independent variable after sequentially adding variables. These all indicate that the estimation results of the ologit model have strong robustness. Specifically, as shown in model 3, rural-urban migrants owning commercial housing can significantly improve their SWB. Therefore, hypothesis 1 is verified.

**Table 3:** Baseline regression results——Ordered logit model.

Variable	Ordered Logit Model		
	model 1	model 2	model 3
Commercial housing	0.140***	0.280***	0.280***
	(2.74)	(4.59)	(4.57)
Gender		0.041	0.040
		(0.69)	(0.67)
Generation		-0.150**	-0.152**
		(-2.36)	(-2.39)
Education		-0.058**	-0.058**
		(-2.25)	(-2.25)
Subjective health status		0.427***	0.426***
		(12.57)	(12.54)
Head		-0.195***	-0.193***
		(-2.92)	(-2.89)
Marital status		0.370***	0.371***
		(5.24)	(5.25)
Residential location		-0.394***	-0.389***
		(-6.19)	(-6.08)
Job type		0.285***	0.287***
		(3.23)	(3.24)
Per-income		0.071***	0.071***
		(4.40)	(4.37)
Per-expenditure		-0.086***	-0.087***
		(-4.17)	(-4.19)
Social security		0.177**	0.179**
		(2.10)	(2.12)
Social attitude		0.462***	0.465***
		(3.82)	(3.84)
PerGDP		-0.068	-0.086
		(-0.89)	(-0.84)
Population scale		-0.111***	-0.122***
		(-2.85)	(-2.99)
Area	No	No	Yes
χ^2^	7.49***	327.69***	327.80***
Pseudo R^2^	0.0005	0.0278	0.0279
Observations	5477	5477	5477

Note: Z-statistics in parentheses *** p<0.01, ** p<0.05, * p<0.1.

Regarding the results of the estimation of control variables, as shown in model 3, most of the variables also have a significant effect on the SWB of rural-urban migrants. (1) Among the demographic variables, compared with the second-generation rural-urban migrants, the first-generation rural-urban migrants have a stronger sense of SWB. The possible reason is that the first-generation rural-urban migrants already gained some capital and contacts in the places they moved to, and are gradually adapting to town life. With the improvement of education level, the SWB of rural-urban migrants shows a downward trend. The reason may be that the higher the level of education, the higher the demands on their quality of life, and thus the higher the threshold of their perceived happiness. This is consistent with the findings of Knight and Gunatilaka [47]; In addition, rural-urban migrants with higher subjective evaluations of their own health have a stronger sense of SWB, in line with the findings of Jiang et al. [52]. Unlike other studies [47, 52], this study did not find that gender has a significant effect on the SWB of rural-urban migrants; (2) In terms of household characteristics variables, the head of the household has a poorer sense of SWB compared to other household members. As the head of the household, the responsibilities of the family tend to put more pressure on him/her, which reduces the perception of SWB. Rural-urban migrants who are currently in a married status have a stronger sense of SWB than those in other marital statuses, in line with existing research [52, 53]. In addition, compared with those that live in towns, rural-urban migrants whose families currently live in rural areas are happier. This may be due to greater financial pressure on families living in towns; (3) Among the socio-economic characteristics variables, the higher the income and the lower the daily expenses, the greater the SWB of rural-urban migrants. Those working in governmental department and organization/public institution or SOE/state holding enterprise have a greater sense of SWB than other job types; (4) Regarding rural-urban migrants’ social security measured by the availability of social medical insurance, the results show that access to social medical insurance significantly enhances the SWB of rural-urban migrants, in line with the findings of Cheng and Hua [54]; (5) In terms of social attitude of rural-urban migrants, measured by the trust level in strangers, it is found that the higher the level of trust, the higher the SWB of rural-urban migrants, which is consistent with the research of Yip et al. [55]; (6) In the control variables for provincial (municipality) characteristics, perGDP and population scale are both negatively correlated with SWB, which may be because the more developed areas are, the faster the pace of life and the higher the cost of living, thus affecting the SWB of rural-urban migrants. But the effect of perGDP is not significant.

### 4.2 Robustness checks

#### 4.2.1 Robustness tests based on OProbit and OLS (ordinary least square) model

The regression estimation of the dependent variable as an ordinal variable can use the ologit model and oprobit model. The main difference between the two is that the dependent variable obeys different distributions, with the logit model following a standard logistic distribution, and the probit model following a standard normal distribution. Angrist and Pischke believe that under the condition of accurate model setting, whether OLS or ordinal probability model (Ologit/Oprobit) is used for regression, there is no difference in results [64]. And Ferrer-i-Carbonell and Frijters [65] also pointed out that there is no significant difference in the coefficients of regression results and the sign and significance of marginal effects between the OLS model and the Ordered Probit/Logit model. In addition, with the number of grades increases (5 and above), the difference between Logistic regression and OLS regression becomes smaller and smaller [66]. Therefore, the Ologit estimation results were tested for robustness using the Oprobit model and OLS model. Consistent with the baseline regression, the independent variable, control variables and regional dummy variable are added in the model in turn, and the regression results are shown in Table 4. Comparing model 6 and model 9 with model 3 reveals that the estimation results of the three models are generally consistent, indicating that the empirical results are robust and credible.

**Table 4:** Robustness test based on adjusted models.

Variable	Ordered Probit Model			OLS Model		
	model 4	model 5	model 6	model 7	model 8	model 9
Commercial housing	0.093***	0.161***	0.161***	0.077***	0.119***	0.118***
	(3.13)	(4.64)	(4.63)	(3.48)	(4.72)	(4.69)
Gender		0.025	0.024		0.019	0.019
		(0.73)	(0.71)		(0.77)	(0.74)
Generation		-0.089**	-0.090**		-0.060**	-0.061**
		(-2.44)	(-2.46)		(-2.30)	(-2.32)
Education		-0.028*	-0.028*		-0.018*	-0.018*
		(-1.88)	(-1.88)		(-1.66)	(-1.66)
Subjective health status		0.237***	0.236***		0.171***	0.170***
		(12.45)	(12.42)		(12.37)	(12.34)
Head		-0.115***	-0.114***		-0.084***	-0.083***
		(-2.99)	(-2.96)		(-2.98)	(-2.95)
Marital status		0.224***	0.224***		0.172***	0.172***
		(5.60)	(5.60)		(5.80)	(5.79)
Residential location		-0.219***	-0.217***		-0.155***	-0.152***
		(-5.99)	(-5.88)		(-5.82)	(-5.70)
Job type		0.163***	0.163***		0.114***	0.115***
		(3.13)	(3.14)		(3.08)	(3.09)
Per-income		0.044***	0.043***		0.033***	0.033***
		(4.68)	(4.65)		(4.67)	(4.65)
Per-expenditure		-0.049***	-0.049***		-0.035***	-0.035***
		(-4.07)	(-4.09)		(-4.04)	(-4.05)
Social security		0.087*	0.088*		0.062*	0.063*
		(1.82)	(1.83)		(1.78)	(1.80)
Social attitude		0.236***	0.237***		0.151***	0.152***
		(3.31)	(3.32)		(2.97)	(2.99)
PerGDP		-0.040	-0.051		-0.024	-0.031
		(-0.90)	(-0.87)		(-0.76)	(-0.74)
Population scale		-0.063***	-0.070***		-0.044***	-0.048***
		(-2.83)	(-3.00)		(-2.72)	(-2.89)
Area	No	No	Yes	No	No	Yes
χ^2^/F	9.83***	323.20***	323.54***	12.08***	22.49***	19.87***
Pseudo R^2^/ R^2^	0.0007	0.0272	0.0273	0.0021	0.0616	0.0619
Observations	5477	5477	5477	5477	5477	5477

Note: Z-statistics in parentheses (Ordered Logit Model and Ordered Probit Model), t-statistics in parentheses (OLS Model) *** p<0.01, ** p<0.05, * p<0.1.

#### 4.2.2 Robustness tests based on adjusted samples

The paper further adjusts the sample size to test the regression results. As shown in Table 5, ologit regressions are conducted on a randomly selected 80% sample, a sample in the age range of 27–45, and a sample with an annual per capita income of 0.90–3.08 (ten thousand). The upper and lower bounds of sample selection for the variable of age and annual income per capita are the upper and lower quartile values of the data for that variable. The comparison reveals that the calculated results are consistent with the findings of the analysis above, indicating that the study findings are relatively robust (due to space constraints, only the regression results for the independent variable are presented).

**Table 5:** Ordered logit regression model for adjusted samples.

Variable	Ordered Logit Model		
	model 10	model 11	model 12
	80% sample	Sample aged 27–45 years	Sample with an annual per capita income of 0.9–3.08 (ten thousand)
Commercial housing	0.286***	0.249***	0.276***
	(4.15)	(3.00)	(3.20)
Control variables	Yes	Yes	Yes
Area	Yes	Yes	Yes
χ^2^	285.89***	175.97***	200.27***
Pseudo R^2^	0.0300	0.0281	0.0330
Observations	4382	3041	2739

Notes: Z-statistics in parentheses *** p<0.01, ** p<0.05, * p<0.1.

### 4.3 Endogeneity discussion results

#### 4.3.1 Correcting selection bias——Propensity Score Matching (PSM)

**Fig 2:**
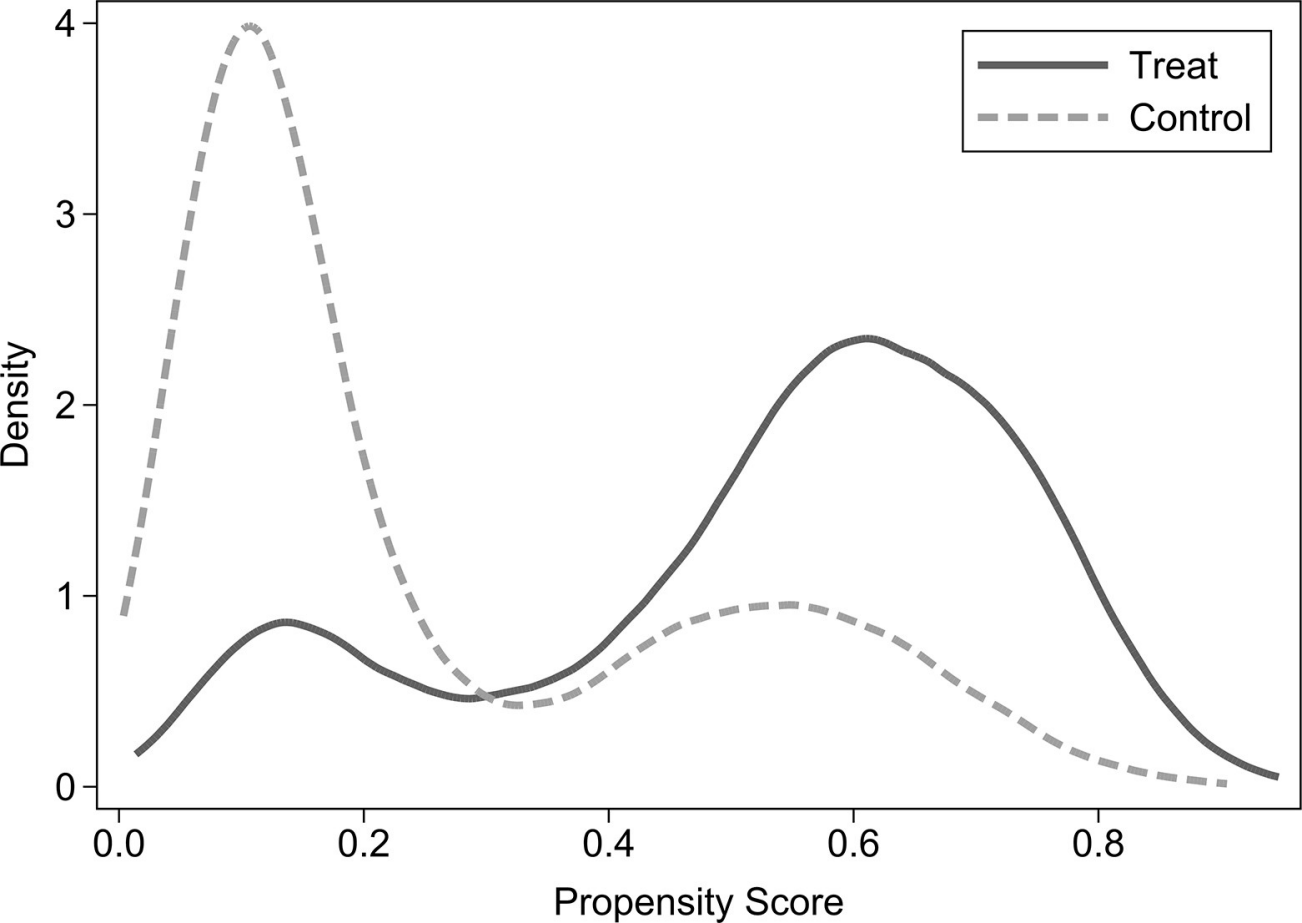
Kernel density distributions before matching.

**Fig 3:**
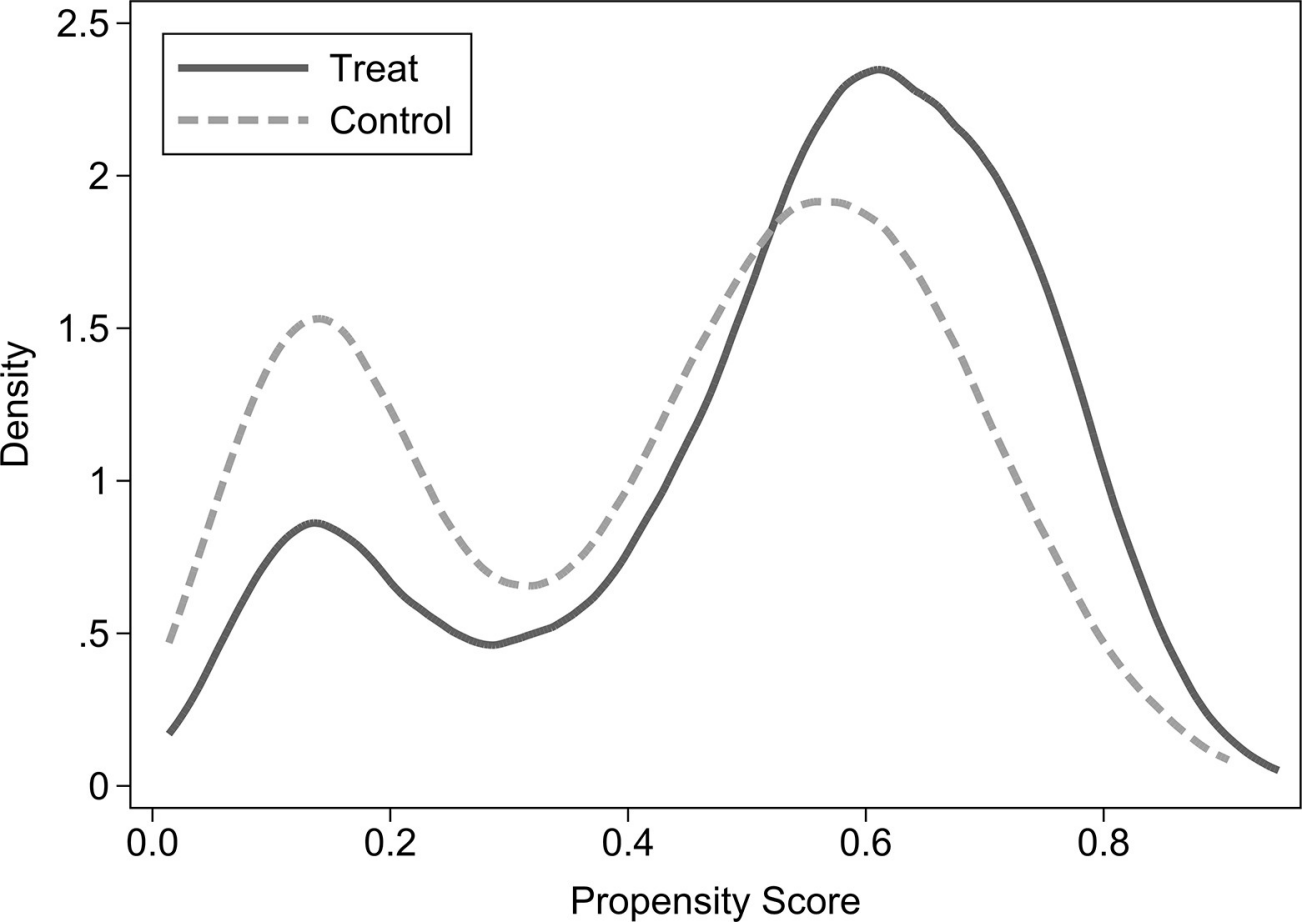
Kernel density distributions after matching.

The PSM method is used to correct for self-selection bias in the “ownership of commercial housing” of rural-urban migrants in order to reduce the endogeneity issues in the sample estimation. To improve the matching efficiency, this paper uses kernel matching method for propensity score matching (PSM). The balanced test of the matched results is shown in Table 6, which shows that the standardized deviations (%bias) of all variables after matching are less than 5% and all t-test results are insignificant at the 10% statistical level. This shows that the matched results passed the balanced test. Therefore, the original hypothesis of no systematic difference between the treated and control groups is accepted. Moreover, Figs 2 and 3 show the kernel density functions of the individual propensity score values of the treated group and the control group before and after matching. It shows that the difference between the kernel density equation curves of the two groups is significantly reduced after matching, and the trend tends to be consistent. So the common support hypothesis is satisfied. The above results show that the PSM method does weaken the distribution differences of the independent variable between the two groups, and the matching process is successful.

**Table 6:** Balance test results.

Variable	Unmatched Matched	Mean		%bias	%reduct |bias|	t	P>|t|
		Treated	Control				
Gender	U	0.586	0.607	-4.3	86.9	-1.53	0.126
	M	0.586	0.583	0.6		0.18	0.861
Generation	U	0.522	0.596	-15.0	84.4	-5.34	0.000
	M	0.520	0.509	2.3		0.72	0.469
Education	U	3.578	3.195	31.5	86.2	11.30	0.000
	M	3.573	3.520	4.3		1.33	0.184
Subjective health status	U	3.933	3.754	20.1	98.6	7.06	0.000
	M	3.931	3.933	-0.3		-0.09	0.926
Head	U	0.395	0.259	29.4	98.2	10.59	0.000
	M	0.395	0.397	-0.5		-0.16	0.875
Marital status	U	0.846	0.696	36.2	96.5	12.39	0.000
	M	0.845	0.840	1.3		0.45	0.654
Residential location	U	0.829	0.357	109.7	99.9	37.58	0.000
	M	0.829	0.829	-0.1		-0.02	0.981
Job type	U	0.093	0.094	-0.2	-1763.4	-0.08	0.936
	M	0.093	0.081	4.2		1.35	0.178
Per-income	U	9.979	9.254	43.4	91.4	15.35	0.000
	M	9.972	9.910	3.7		1.30	0.192
Per-expenditure	U	6.907	5.962	76.5	96.7	26.01	0.000
	M	6.898	6.930	-2.6		-0.90	0.371
Social security	U	0.875	0.905	-9.4	85.4	-3.40	0.001
	M	0.876	0.872	1.4		0.41	0.685
Social attitude	U	0.058	0.054	1.6	-174.7	0.58	0.564
	M	0.057	0.068	-4.5		-1.32	0.186
PerGDP	U	10.999	10.931	19.8	92.0	7.07	0.000
	M	10.999	11.005	-1.6		-0.47	0.638
Population scale	U	8.515	8.545	-4.4	82.9	-1.55	0.121
	M	8.518	8.523	-0.7		-0.23	0.820
Central region	U	0.262	0.304	-9.4	56.7	-3.31	0.001
	M	0.261	0.243	4.1		1.32	0.188
Western region	U	0.258	0.282	-5.5	74.0	-1.94	0.052
	M	0.257	0.263	-1.4		-0.45	0.652

As shown in Table 7, different matching methods are used to measure the ATT values of the two groups of samples after matching to ensure the reliability of the estimation results. According to the results of the ATT, after eliminating the observable systematic differences between the two sample groups, the ownership of commercial housing by rural-urban migrants has a significant positive effect on their SWB at the 1% statistically level. Specifically, the maximum value of ATT for local linear regression matching is 0.148 and the minimum value of ATT for 1-nearest neighbor matching and radius matching is 0.105. Although the values of ATT vary slightly under different matching methods, the estimated results are generally consistent, indicating that the measurement results are robust [67].

**Table 7:** Estimation results of the PSM method.

Matching method	Match	Treated	Control	ATT	SD	T
	before	3.889	3.812	0.077***	0.023	3.36
Kernel matching	After	3.887	3.743	0.144***	0.029	4.90
1-nearest neighbor	After	3.887	3.782	0.105***	0.038	2.76
4-nearest neighbor	After	3.887	3.769	0.118***	0.032	3.70
Radius matching (radius = 0.05)	After	3.887	3.782	0.105***	0.038	2.76
Local linear regression matching	After	3.887	3.740	0.148***	0.038	3.86

Note: Default values are used for both kernel matching and local linear regression matching.

#### 4.3.2 Instrumental variable regression——Conditional Mixed Process (CMP)

Apart from selection bias, this study may still face endogeneity problems such as omitted variables and reverse causality. To this end, this study combines instrumental variables and CMP method for analysis.

Table 8 reports the regression results based on the CMP instrumental variables approach, with model 14 and model 16 controlling for regional variables in turn. Overall, the instrumental variable in the first stage are significantly positively correlated with commercial housing, whether using the ownership rate of commercial housing in each prefecture-level cities (IV1) as the instrumental variable or further using rural data (IV2) as the instrumental variable, the estimation results of the second stage show that owning commercial housing contributes to improving the SWB of rural-urban migrants. As a consequence, after accounting for endogeneity, owning commercial housing still has a significant positive effect on the SWB of rural-urban migrants.

**Table 8:** Estimated results based on CMP method.

Variable	Conditional Mixed Process (CMP) Ordered Probit OLS							
	model 13		model 14		model 15		model 16	
	cmp_ oprobit	cmp_ probit	cmp_ oprobit	cmp_ probit	cmp_ oprobit	cmp_ probit	cmp_ oprobit	cmp_ probit
	SWB	Commercial housing	SWB	Commercial housing	SWB	Commercial housing	SWB	Commercial housing
Commercial housing	1.035***		0.977***		1.068***		1.083***	
	(8.71)		(7.25)		(9.25)		(9.56)	
IV1		3.889***		4.113***				
		(8.19)		(8.36)				
IV2						28.388***		26.708***
						(6.71)		(6.21)
atanhrho_12	-0.597***		-0.549***		-0.623***		-0.636***	
	(-5.99)		(-5.08)		(-6.27)		(-6.44)	
Control variables	Yes		Yes		Yes		Yes	
Area	No		Yes		No		Yes	

Note: Z-statistics in parentheses *** p<0.01, ** p<0.05, * p<0.1.

The results of the CMP model report the endogeneity test parameter atanhrho_12 in addition to the correlation. When the atanhrho_12 parameter is significant, it can be considered that the baseline model has endogeneity. As shown in Table 8, the independent variable of commercial housing in this study do have endogenous issues. Moreover, in order to further test the validity of the instrumental variables, this study refers to the existing literature [68], and borrows the weak instrumental variable test method of the linear model to test the model 13 to model 16 in Table 8. The results are shown in Table 9. The F-statistics for model 13 to 16 are all greater than 10 and are all significant at the 1% statistical level. Also, the results of the Wald test at a nominal significance level of 5% show that the minimum eigenvalue statistic is much larger than the critical value of 10%. Based on this, the issue of weak instrumental variables can be ruled out.

**Table 9:** Weak instrumental variable test.

	model 13	model 14	model 15	model 16
F-statistics	85.0332***	91.5908***	58.0379***	46.5794***
Minimum eigenvalue statistic	88.2641	95.0852	58.5154	46.3861
Critical value of 10%	16.38	16.38	16.38	16.38
Critical value of 15%	8.96	8.96	8.96	8.96

## 5 Further discussion

### 5.1 Heterogeneity analysis

From the above baseline regression results we found that owning commercial housing significantly enhances the SWB of rural-urban migrants. However, the above results only represent the average effect on the SWB of rural-urban migrants, and do not take into account the intra-group differences among rural-urban migrants. Therefore, this study also analyses the heterogeneity of the effect of commercial housing on the SWB of rural-urban migrants from the perspective of intergenerational, regional and housing acquisition time. Specifically, the intergenerational classification includes the first generation and second generation. Consistent with the conventional definition, the first generation are those born before 1980, and the second generation are those born in the 1980s and after. Using the classification standard of the National Bureau of Statistics, the 29 provinces in CHFS are divided into eastern, central and western regions. In addition, housing prices in China remained comparatively stable before 2004 when the open auction for the transfer of land use rights was introduced by the government, but it began to soar after 2004 [69]. So we take 2004 as the dividing line to explore whether there is a difference in the impact of owning commercial housing on the SWB of rural-urban migrants before and after the change of housing price.

As shown in Table 10, overall, the strengthening effect of commercial housing ownershipon the SWB of rural-urban migrants remains, which is generally consistent with the results of the baseline regression. Further from an intergenerational perspective, compared to the second generation rural-urban migrants, commercial housing ownership has a more pronounced reinforcing effect on the SWB of the first generation rural-urban migrants. They have a deeper sense of home ownership. As their children are of school or marriageable age, owning commercial housing in town not only provides better educational resources for their children, but is also a key bargaining chip for entering into marriage. For the second generation, the rising housing prices put increasing difficulty to own commercial housing, at the same time, with the rapid development of the rental market, renting private houses has become the main form of residence for them when they first arrived in a new place (Calculated from the 2017 China Migrants Dynamic Survey (CMDS) data.).

**Table 10:** Results of the heterogeneity analysis.

Variable	Ordered Logit Model						
	model 17	model 18	model 19	model 20	model 21	model 22	model 23
	The first generation	The second generation	Eastern	Central	Western	Time(year) < = 2004	Time(year) >2004
Commercial housing	0.298***	0.239***	0.380***	0.289**	0.142	0.402***	0.186**
	(3.35)	(2.76)	(4.25)	(2.20)	(1.23)	(3.80)	(2.11)
Control variables	Yes	Yes	Yes	Yes	Yes	Yes	Yes
Area	Yes	Yes	——	——	——	Yes	Yes
χ^2^	182.72***	164.42***	131.92***	106.49***	131.96***	178.28***	180.36***
Pseudo R^2^	0.0350	0.0251	0.0283	0.0298	0.0399	0.0350	0.0302
Observations	2356	3121	2397	1583	1497	2166	2792

Note: Z-statistics in parentheses *** p<0.01, ** p<0.05, * p<0.1.

From a regional perspective, owning commercial housing has a statistically significant positive impact on the SWB of rural-urban migrants in the eastern and central regions, but not in the western. The impact is higher in the east than in the center region. According to the interpretation of the National Development and Reform Commission, the division of China ’s eastern, central and western regions is a policy division, not an administrative division, nor a geographical concept division. Therefore, the eastern part refers to the provinces and cities that first implemented the coastal opening policy and had a high level of economic development; the central region refers to the economically sub-developed areas, while the western region refers to the economically underdeveloped areas. Furthermore, the level of economic development determines the level of housing prices, the quality of public services, employment opportunities, educational resources and migration willingness. Therefore, compared with the western region, the eastern and central regions are the main gathering places of population migration, and owning commercial housing in the central and eastern regions can better reflect their core competitiveness, symbolize their social status, and thus enhance their SWB, and this effect is most obvious in the eastern region.

From the perspective of housing acquisition time, the effect of owning commercial housing on the SWB of the rural-urban migrants who obtained housing before 2004 is more pronounced. In other words, the happiness effect is more significant before the housing price changes. This result is not difficult to understand, rising housing prices lead to higher housing costs for rural-urban migrants to obtain commercial housing, which impacts their perception of SWB to some extent. However, before the rise of housing price, the housing price-to-income ratio was relatively low. At that time, acquiring commercial housing not only had a lower cost, but also brought the corresponding wealth effect due to the subsequent appreciation of housing, both of which enhanced their SWB.

### 5.2 Effect mechanism analysis

#### 5.2.1 Mediating effect analysis

Table 11 reports the regression results of the stepwise method to verify the mediation effect. It can be seen from model 24 to model 27 that owning commercial housing significantly increases the household housing assets of rural-urban migrants, and housing assets have a significant positive correlation with the SWB of rural-urban migrants. However, it is still not possible to judge whether owning commercial housing affects the SWB by affecting the household housing assets.

**Table 11:** Estimated results of the mediating effect model.

Variable	Ordered Logit Model		Ordered Probit Model		OLS Model	
	model 24	model 25	model 26	model 27	model 28	model 29
	Housing assets	SWB	Housing assets	SWB	Housing assets	SWB
Commercial housing	3.159***	0.240***	3.159***	0.138***	3.159***	0.102***
	(20.09)	(3.69)	(20.09)	(3.76)	(20.09)	(3.82)
Housing assets		0.012**		0.007**		0.005**
		(2.16)		(2.32)		(2.32)
Control variables	Yes	Yes	Yes	Yes	Yes	Yes
Area	Yes	Yes	Yes	Yes	Yes	Yes
χ^2^/F	63.48***	332.18***	63.48***	329.70***	63.48***	19.12***
Pseudo R^2^/ R^2^	0.1418	0.0282	0.1418	0.0277	0.1418	0.0629
Observations	5477	5477	5477	5477	5477	5477

Note: Z-statistics in parentheses (Ordered Logit Model and Ordered Probit Model), t-statistics in parentheses (OLS Model) *** p<0.01, ** p<0.05, * p<0.1.

Consequently, the influence effects of the ologit model and oprobit model are decomposed according to the KHB method mentioned in the previous section, as shown in Table 12. From Table 12, it can be seen that the mediating effects of the household housing assets of rural-urban migrants in both models are all significantly positive, thus indicating that the variable of household housing assets is a significant pathway through which the commercial housing affects the SWB of rural-urban migrants. Furthermore, the total effect and direct effect are also significantly positive, which again verifies the previous conclusion. That is to say, commercial housing ownership has a positive impact on the SWB of rural-urban migrants. The ratio of the mediating effect to the total effect coefficient shows that the mediating effect accounts for 31.1% of the total effect, meaning that 31.1% of the effect of commercial housing ownership on the SWB of rural-urban migrants is achieved by increasing their housing assets.

**Table 12:** Mediation test——KHB method.

Mediator Variable	Ordered Logit Model	Ordered Probit Model
Total effect	0.0169***	0.0102***
	(3.28)	(3.43)
Direct effect	0.0116**	0.0071**
	(2.17)	(2.33)
Mediating effect	0.0052***	0.0030***
	(3.67)	(3.67)

Note: Z-statistics in parentheses *** p<0.01, ** p<0.05, * p<0.1.

In addition, we take SWB as a continuous variable and then perform OLS regression on the mediating effect of housing assets. According to the sequence of stepwise tests of the mediating effect model, the regression coefficient of commercial housing on SWB is significantly positive from model 9, the regression coefficient of commercial housing on housing assets is significantly positive from model 28, and then the regression coefficient of housing assets on SWB is significantly positive from model 29, which indicates that the BK stepwise regression passes the test in turn. This proves that the variable of housing assets is one of the channels through which commercial housing affects the SWB of rural-urban migrants. At the same time, Bootstrap method is used to test the mediating effect again. 500 Bootstrap samples are drawn by random repeated sampling, and the estimated value of 500 coefficient products is obtained. As shown in Table 13 for the indirect effect (bs_1), 0 is not included in the 95% confidence interval of the mediating effect value, indicating that the coefficient product is significant and the mediating effect is established. The confidence interval is on the left side of 0, indicating that the mediation effect is positive.

**Table 13:** Mediation test——Bootstrap method.

	Observed	Bootstrap	Z	P>|z|	Normal-based	
	Coef.	Std. Err.			[95% Conf. Interval]	
_bs_1	0.0166354	0.007109	2.34	0.019	0.0027019	0.0305689
_bs_2	0.101582	0.0269785	3.77	0.000	0.0487051	0.154459

The above results robustly verify the research hypothesis 2. That is, the asset attribute of commercial housing is an important path affecting the SWB of rural-urban migrants, and housing has a certain wealth effect. In addition, to explore the regional differences in the mediating effect of housing assets, we further analyse the sample of rural-urban migrants in the east, central and west respectively using the KHB method, and the regression results are shown in Table 14.

**Table 14:** Regional differences in the mediating effect of housing assets.

Mediator Variable	Ordered Logit		
	Eastern	Central	Western
Total effect	0.0166**	0.0293***	0.0031
	(2.26)	(2.83)	(0.29)
Direct effect	0.0067	0.0264**	0.0005
	(0.86)	(2.52)	(0.05)
Mediating effect	0.0099***	0.0028*	0.0025
	(3.61)	(1.67)	(1.12)

The mediating and total effects of housing assets in the eastern region are both significantly positive and the direct effect is not significant, indicating that housing assets play a fully mediating role in the eastern region. Although the results may be biased due to sample size, it is sufficient to show that the asset attribute of commercial housing in the eastern region is the main mediator of rural-urban migrants’ SWB. The mediating effect was significant in the central region, with a share of around 10%, which was lower than the share of mediating effect in the total sample. In the Western region, housing assets did not have a mediating effect. This finding is consistent with the actual situation in China and similar to the results of the heterogeneity analysis above. The eastern coastal region has a developed economy, more employment opportunities, higher levels of public services than the central and western regions, and relatively higher housing prices, and the fixed-asset effect of commercial housing in the eastern region brings a strong sense of SWB to rural-urban migrants.

#### 5.2.2 Moderating effect analysis

To ensure the robustness of the test results, three models of Ologit, Oprobit and OLS are used to analyse the moderating effect of commercial housing and debt. The regression results are shown in Table 15.

**Table 15:** Estimated results of the moderating effect model.

Variable	Ordered Logit Model		Ordered Probit Model		OLS Model	
	model 30	model 31	model 32	model 33	model 34	model 35
Commercial housing	0.287***	0.165**	0.167***	0.100**	0.124***	0.075**
	(4.59)	(2.00)	(4.71)	(2.14)	(4.81)	(2.23)
Household debt	-0.032	-0.118*	-0.028	-0.074*	-0.023	-0.057**
	(-0.60)	(-1.73)	(-0.93)	(-1.89)	(-1.06)	(-1.97)
Interaction term		0.238**		0.129**		0.094**
		(2.20)		(2.09)		(2.11)
Control variables	Yes	Yes	Yes	Yes	Yes	Yes
Area	Yes	Yes	Yes	Yes	Yes	Yes
χ^2^/F	328.10***	332.56***	324.62***	328.84***	18.84***	18.10***
Pseudo R^2^/ R^2^	0.0279	0.0283	0.0273	0.0277	0.0621	0.0628
Observations	5477	5477	5477	5477	5477	5477

Note: Z-statistics in parentheses (Ordered Logit Model and Ordered Probit Model), t-statistics in parentheses (OLS Model) *** p<0.01, ** p<0.05, * p<0.1.

First, the results of model 3, model 6 and model 9 above suggest that commercial housing ownership can significantly improve the SWB of rural-urban migrants. Then, the moderator variable of debt is added to model 30, model 32 and model 34, and the interaction effect of commercial housing and debt is added to model 31, model 33 and model 35 in turn. We find that debt has a significant negative effect on the SWB of rural-urban migrants, while the coefficient of the interaction term is significantly positive. So we believe that debt plays a certain moderating role between commercial housing and the SWB of rural-urban migrants. In other words, debt can reinforce the positive effect of commercial housing on the SWB of rural-urban migrants, and Hypothesis 3 is verified.

Why does debt instead increase the positive impact of owning commercial housing on the SWB of rural-urban migrants? In China, housing is the foundation of "living and working in peace and contentment", especially for rural-urban migrants who come to cities or towns from rural areas, the most basic motivation for them to own commercial housing is to have a place to live and settle down here, to enjoy high-quality public services such as medical care and education, and to improve their competitiveness in the marriage market. Owning commercial housing is an important part of China’s urbanization process. At the same time, most of the rural-urban migrants in this study have only one commercial housing and there are no other motives such as property speculation. Therefore, in this case, debt has a positive moderating effect on the relationship between owning commercial housing and the SWB of rural-urban migrants, which we interpret to mean that whether it is the housing loan or other borrowing that rural-urban migrants takes out in order to own commercial housing, these debts to some extent smooth out the consumption dilemmas arising from housing expenditure, thus positively moderating their SWB. Although owning commercial housing may increase household debt, it also means that these rural-urban migrants have relatively strong economic strength and can afford the corresponding debt, and more importantly, owning commercial housing also symbolizes the change in status and the realization of the "urban dream". Accordingly, this logic holds true for the relationship between owning commercial housing and the SWB of rural-urban migrants. This further supplements the research findings of Zheng et al. that there is no evidence of a correlation between financial constraints and SWB decline among those with homeownership [7].

### 5.3 Extensibility analysis

It has been confirmed above that rural-urban migrants whose families currently live in rural areas are more happier (Rural-urban migrants whose families currently live in rural areas means that the main economic and social activities of rural-urban migrants’ families still occur in rural areas. For example, in a family, the male rural-urban migrant works in cities to make money, while other family members still live in rural areas. He returns to the countryside to reunite with his family only during the Spring Festival, with strong mobility between urban and rural areas. That is to say, family-based urban migration has not yet been completed.). But this result is only an overall effect and does not distinguish impact for rural-urban migrants who own commercial housing versus those who do not. Therefore, this study further explores the heterogeneous effect of the current residential location of households in these two groups on the SWB of rural-urban migrants. It can be seen from Table 16 that whether or not one owns commercial housing, the location of the family’s current residence in towns has a significant negative effect on the SWB of rural-urban migrants. However, compared to rural-urban migrants who do not own commercial housing, the reduction effect of SWB is smaller for rural-urban migrants who own. This also indirectly supports the core hypothesis of this research that owning commercial housing can improve the SWB of rural-urban migrants. Thus, hypothesis 4 is confirmed.

**Table 16:** Results of the extensibility analysis.

Variable	Ordered Logit Model	
	model 36	model 37
	Owning commercial housing	No commercial housing
Current residential location of the family	-0.358***	-0.403***
	(-2.76)	(-5.45)
Other control variables	Yes	Yes
Area	Yes	Yes
χ^2^	107.89***	255.67***
Pseudo R^2^	0.0270	0.0323
Observations	1945	3532

Note: Z-statistics in parentheses *** p<0.01, ** p<0.05, * p<0.1.

So why has owning commercial housing failed to reverse rural-urban migrants’ perceptions of the SWB when their families live in towns? The possible reason is that the cost of urban living is still the primary problem of rural-urban migration, compared with living in rural areas, family-oriented urban migration will bring huge economic burdens. In addition, their social interactions are usually on blood and local ties, out of the familiar living environment will make them feel insecure, and it is relatively difficult to reconstruct the social network in cities and towns, rural-urban migrants’ families have not really integrated into urban life. As a result, even with commercial housing, their families live in rural areas makes rural-urban migrants feel happier. The family-based urbanization process still has a long way to go.

## 6 Conclusions and implications

Based on the data of China Household Finance Survey in 2017, we use Ordered Logit model and take rural-urban migrants as the research object to explore the relationship between owning commercial housing and SWB of low-income groups, and that the heterogeneous impact on the intergenerational, regional and housing acquisition time, and then explore the underlying mechanism of its impact from the perspective of housing assets and household debt. Finally, we further expand the research on the current living location of families, commercial housing and the SWB of rural-urban migrants. The results show that: Firstly, commercial housing significantly enhances the SWB of rural-urban migrants. The findings remain robust after replacing the model, adjusting the sample size, correcting for sample selectivity bias using propensity score matching (PSM), and controlling for potential endogeneity bias by combining instrumental variables and CMP estimation method. It is proved that the positive effect of commercial housing on SWB still exists in low-income groups; Secondly, the effect of owning commercial housing on the SWB of the first generation rural-urban migrants, rural-urban migrants in the eastern and central regions and those who obtained housing before 2004 is more pronounced. This result is consistent with China’s economic development level and development process; Thirdly, commercial housing acts on the SWB of rural-urban migrants through the mediating effect of housing assets, and the mediating effect accounts for 31.1% of the total effect, especially in the eastern region, housing assets act as the main mediation. And the household debt acts as a positive moderator between commercial housing and the SWB of rural-urban migrants. In other words, the SWB of rural-urban migrants with commercial housing is not reduced by household debt; Fourthly, even with commercial housing, rural-urban migrants whose families are currently living in rural areas still have a stronger sense of SWB. The cost of urban living is still the primary problem of rural-urban migration, the reconstruction of social network is also difficult. The family-based urbanization process still has a long way to go.

Most of the existing studies on housing and the SWB have focused on overall residents or urban residents, and few researchers have explored the relationship between the housing and the SWB of rural-urban migrants and its internal mechanism. This study contributes to the literature by focusing on rural-urban migrants with empirical evidence.The results of this study also have certain policy implications for solving the housing problem of rural-urban migrants:

First, commercial housing, as an important housing asset, has a significant effect on the SWB of rural-urban migrants. Moreover, Chinese residents’ SWB is higher when the local government promises greater dedication to housing affordability improvement [70]. Therefore, for those rural-urban migrants who have the will and ability to own commercial housing in towns, the government should encourage and support their purchase behavior, and adopt various forms of housing preferential policies such as first purchase and then subsidy, quota subsidy, tiered settlement, direct subsidy to households, etc. In addition, the form of land transfer should be innovated to promote the construction of a unified urban and rural land transfer market, for example, try to establish a correlation mechanism between rural homesteads and urban housing, and give reasonable urban housing subsidies to rural-urban migrants who are willing to withdraw from homestead, so as to facilitate the early realization of their "urban dream" and improve their sense of SWB; Second, regional differences-owning commercial housing can significantly improve migrants’ SWB in the eastern and central regions not in the west, which suggests that the west has a weaker housing market. Therefore, the government should increase investment in the western region and play a guiding role in driving social capital to participate in investment, and further strengthen the construction of infrastructure and urbanization in the western region, optimize the investment environment, increase the intensity of opening up, broaden the market and promote economic development. Through these to enhance the competitiveness of the western region and improve the SWB of rural-urban migrants in the western region, so as to alleviate the urban pressure in the eastern and central regions; Third, after owning commercial housing, rural-urban migrants whose families currently live in rural areas still have a greater sense of SWB. This "semi-urbanization" pattern has led to the emergence of a "new dual structure" segmentation phenomenon in towns, with rural-urban migrants and urban residents as the two main bodies [71]. In the meanwhile, the rural areas have given rise to a large number of left-behind children, women and elderly people, which is clearly not conducive to China’s sustained economic growth and long-term social stability. Therefore, it is imperative to advance the reform of the household registration system in a rational and orderly manner, and the residence permit system can be considered for full implementation, no longer dividing household registration by urban and rural standards, and eliminating institutional discrimination. Next, it is very necessary to narrow the gap between urban and rural areas. The construction of public services in rural areas such as the quality of education, the level of grass-roots health services, employment policies and service systems, and the basic medical insurance system should be strengthened to appropriately reduce the pressure of rural population’s outward migration. Finally, rural-urban migrants should be included in the housing security system. Appropriately increase the supply of small and medium-sized affordable housing, capped-price housing and common property housing. At the same time, the entry threshold for rural-urban migrants to apply for security housing should be appropriately reduced, so that they have the opportunity to obtain security housing with property rights, thereby reducing the cost to own housing and enjoying almost the same urban welfare. In addition, it is important to advocate the housing provident fund from "unit embedded" to "social embedded", so as to improve the accessibility of the housing provident fund for rural-urban migrants and reduce their housing repayment pressure.

Although the China household finance survey (CHFS) data used in this study is a national large sample survey data, there are still some shortcomings. First, this paper explores the impact of the independent variable of owning commercial housing on the SWB of rural-urban migrants. However, due to the limitations of data and sample size, no detailed analysis has been carried out on how the location and quantity of their commercial housing affect their SWB, and these issues still require more in-depth research; Second, The timing of the purchase of commercial housing by rural-urban migrants varies, therefore, the 2017 data selected for this study can only observe static information on rural-urban migrants’ housing in that year and cannot capture the dynamic comparison of rural-urban migrants’ SWB before and after owning commercial housing, which is likely to lead to biased estimation results. Hence, in future research, if suitable panel data are available, a more rigorous and detailed study can be considered, which is a direction for future improvement.
